# Physicians' database searches as a tool for early detection of epidemics.

**DOI:** 10.3201/eid0703.010324

**Published:** 2001

**Authors:** V Jormanainen, J Jousimaa, I Kunnamo, P Ruutu

**Affiliations:** Finnish Defence Forces, Helsinki, Finland.

## Abstract

We analyzed retrospectively the use of Physician Desk Reference Database searches to identify epidemics of tularemia, nephropathy, Pogosta disease, and Lyme disease and compared the searches with mandatory laboratory reports to the National Infectious Diseases Register in Finland during 1995. Continuous recording of such searches may be a tool for early detection of epidemics.


### Physicians' Database Searches as a Tool for Early Detection of Epidemics

**Vesa Jormanainen,*† Jukkapekka Jousimaa,‡ Ilkka Kunnamo,§ and Petri Ruutu¶**
*Finnish Defence Forces, Helsinki, Finland; †University of Helsinki, Helsinki, Finland; ‡University of Kuopio, Kuopio, Finland; §Finnish Medical Society Duodecim, Helsinki, Finland; and ¶National Public Health Institute, Helsinki, Finland

We analyzed retrospectively the use of Physician Desk Reference Database searches to identify epidemics of tularemia, nephropathy, Pogosta disease, and Lyme disease and compared the searches with mandatory laboratory reports to the National Infectious Diseases Register in Finland during 1995. Continuous recording of such searches may be a tool for early detection of epidemics.

Epidemics are conventionally recognized through the observation of spatial or temporal clustering of patients with similar illnesses or through laboratory findings indicating unusually high incidence of a specific disease. Surveillance systems based on passive physician reports frequently have low sensitivity and may not be timely. Recognizing an epidemic by detecting clusters of microbiologically verified cases may also involve delays, depending on such factors as the severity and familiarity of the clinical illness, time from the appearance of the first clinical cases of an epidemic to the first appropriate samples taken and transported, and time required for laboratory testing. Samples from geographically scattered cases from the same epidemic may be sent to different laboratories, reducing the sensitivity of detection. A recent study in infectious disease surveillance used as an index the number of cases per week per sentinel medical institution in the area covered by a health center in Japan (1).

Physicians may consult electronic databases for selecting appropriate diagnostic measures or treatment for specific infectious diseases. A computerized set of primary-care guidelines, the Physicians' Desk Reference and Database (PDRD), has been available in Finland since 1989 (2). This database contains structured information on diseases and conditions that are common or important to recognize in primary care. Physicians often seek information about infectious diseases from the PDRD guidelines (3).

We hypothesized that the frequency of physician searches in a popular database could be useful as a complementary tool in early recognition of infectious disease epidemics. In a pilot study, we analyzed retrospectively the feasibility of using surveillance of database searches in the PDRD to identify epidemics of four specific infectious diseases, as recorded in a recently revised National Infectious Diseases Register (NIDR).

### The Study

The PDRD computerized guidelines on CD-ROM are updated three times a year. We collected the frequency of infection guideline-specific searches by producing a log file of all searches. This data-collecting version was piloted in spring 1994 and mailed to all registered PDRD subscribers in 1995 (3). The data for searches were collected from the computer hard disks of individual users during each updating of the program and were mailed to the PDRD maintenance team.

The NIDR in Finland, which was thoroughly revised in 1994, consists of mandatory laboratory reporting of diagnostic findings for more than 70 pathogens or pathogen groups and mandatory physician reporting of 32 microbiologically confirmed infectious diseases (4). Microbiologic laboratories reported approximately 41,000 cases in 1995. From NIDR, we recorded the sampling dates of cases or the date of the report if the sampling date was not available.

We chose four infectious diseases for comparison between PDRD searches and NIDR laboratory reports: tularemia, ICD-10 A21, caused by *Francisella tularensis*; epidemic nephropathy, ICD-10 A98.5, caused by a Puumala virus (a hantavirus); Pogosta disease, ICD-10 A92.8, caused by the Sindbis virus (an arbovirus); and Lyme disease, ICD-10 A69.2, caused by *Borrelia burgdorferi*. In Finland, epidemic nephropathy (758 to 1,305 laboratory-reported infections per year during 1995-1999) and Lyme disease (346 to 538 laboratory reports of *B. burgdorferi* infections per year) are endemic, with pronounced seasonal variation. Laboratory-confirmed cases of tularemia, mostly of the glandular type, and Pogosta disease, an acute syndrome of fever with rash and self-limiting arthritis, are usually rare, but epidemics occur at intervals of several years.

We compared distributions of PDRD searches and NIDR laboratory reports by disease and month for 1995. Because the number of observations was small, we did not test statistical significance between distributions but based our observations on time-frequency graphs produced by calculating each month's proportion of the total number of cases.

PDRD had 477 subscribers in 1995: 48% of the users returned 306 log files on 15,267 searches; 23,083 specific guidelines were read. The five most popular subject areas were dermatology (9% of searches), infectious diseases (8%), cardiology (6%), gastroenterology (6%), and pediatrics (5%). The Lyme disease guideline was the third most frequently

Address for correspondence: Jukkapekka Jousimaa, Silmukuja 4, 80710 LEHMO, Finland; fax: 358-13-2674370; e-mail: jukkapekka.jousimaa@sll.fimnet.fi read, with 144 readings; Pogosta disease was fifth, with 91; epidemic nephropathy eighth, with 87; and tularemia nineteenth, with 68 readings.

Three hundred forty-six laboratory-confirmed *B. burgdorferi* cases were reported to NIDR, distributed throughout the year (incidence 0.68 per 100,000; 95% confidence interval [CI] 0.60-0.75), but peaking in August. A large epidemic of Pogosta disease took place in late summer and autumn 1995. Cases were distributed throughout the country, with 1,310 laboratory-confirmed (incidence 2.56 per 100,000; 95% CI 2.42-2.70). The 888 laboratory-confirmed cases of Puumala virus infection (1.74; 95% CI 1.62-1.85) peaked in late 1995 and occurred throughout Finland. A major epidemic of tularemia, involving 467 laboratory-reported cases (0.91; 95% CI 0.83-1.00) and distributed over central and southern Finland, began in July 1995 and continued until late autumn.

The distributions of PDRD readings and laboratory-reported cases of the four infectious diseases fell into two patterns (Figure). For tularemia (Figure, panel D) and Pogosta disease (Figure, panel C), the PDRD searches and the cases in NIDR rose from a low baseline level, peaked sharply, and then declined in parallel. For epidemic nephropathy (Figure, panel B), the curves followed the same pattern only partly, with PDRD searches peaking twice, the major peak occurring earlier than in NIDR, in the latter half of 1995. For Lyme disease (Figure, panel A), PDRD search data had a peak well before that of NIDR reports during June to September.

### Conclusions

The temporal correlation observed between the distribution of database searches and laboratory reports for Pogosta disease and tularemia supports the concept that continuous monitoring of database searches for specific infections could be a novel tool for surveillance and detection of epidemics. We are not aware of previous reports of this application for electronic desk reference database searches. For this investigation, we used retrospectively collected logs of CD-ROM–based searches from computers of physicians who used the widely distributed and popular electronic guideline database. The statistics on searches were created automatically into log files without active input by the physicians, and the users had free access to the data they were providing. Although only a few users expressed negative attitudes towards data collection, fewer than half of subscribers returned the data.

Monitoring database searches has the potential to provide timely recognition of an epidemic, as physicians are more likely to use searches to seek guidance for the diagnostics and management of the first patients of an unusual cluster, even before an order for laboratory work is given. Another potential benefit of monitoring database searches at a central facility is that it consolidates consultations from different geographic areas, making it possible to detect subtle changes in widely distributed cases.

Access through the Internet to databases such as the PDRD provides an opportunity to monitor the searches for specific topics. In 1999, the PDRD had 3,500 subscribers throughout Finland, a sevenfold increase from 1995, the year

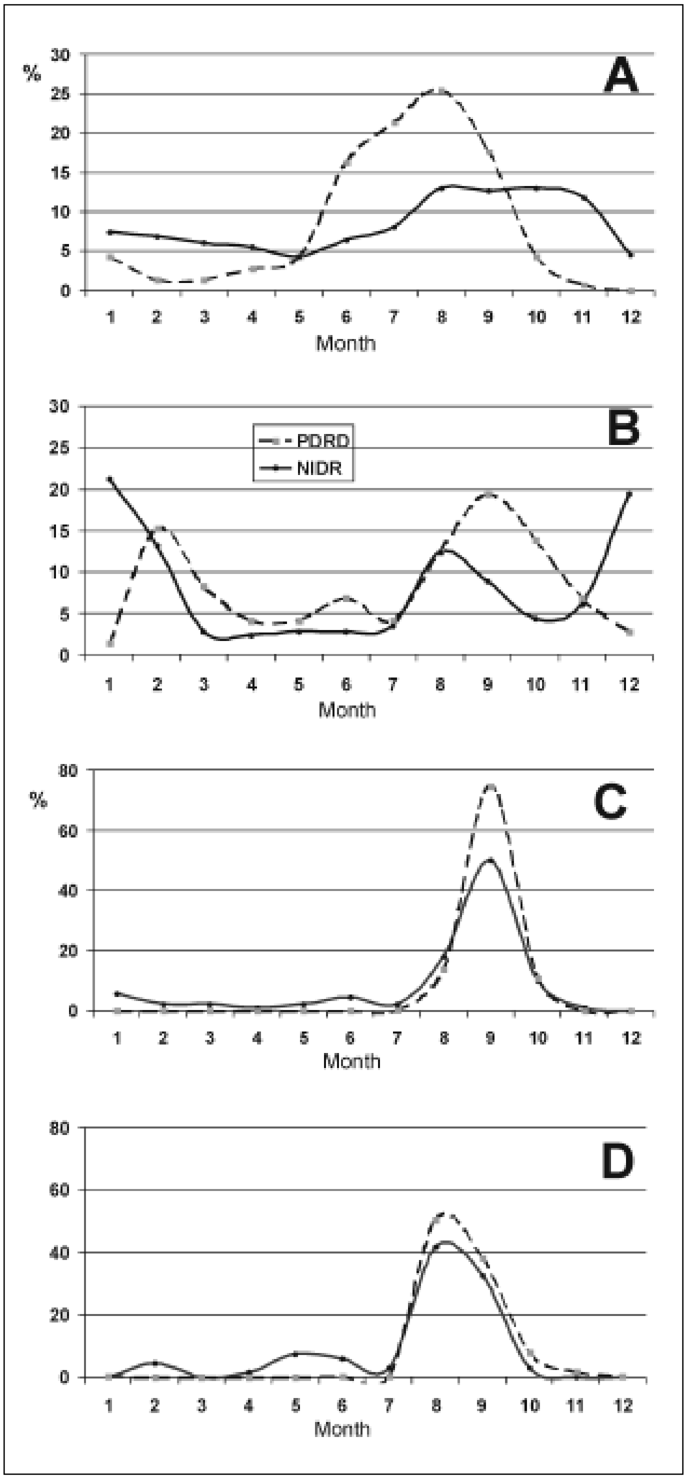

Figure. Laboratory reports to the National Infectious Diseases Register compared with searches on the Physicians' Desk Reference and Database, Finland, 1995. Panel A, *Borrelia burgdorferi* and Lyme disease; panel B, Puumala virus and epidemic nephropathy; panel C, Sindbis virus and Pogosta disease; panel D, *Francisella tularensis* and tularemia.

of this study. An Internet version of the PDRD guidelines (now renamed Evidence-Based Medicine Guidelines) (5), which collects a log file of all searches to the database, was introduced in October 2000. In the future, log files will automatically be sent from CD-ROM users, with their permission. As no patient data are transmitted, no concerns about confidentiality can arise.

Reference databases are likely to be used by clinicians more frequently for syndromes or suspected infections if the physician encounters the specific problem infrequently, e.g., infectious diseases with low transmission levels between epidemics. This observation is supported by the close correlation between the distributions in PDRD searches and NIDR reports of tularemia, epidemics of which have not occurred annually, and Pogosta disease, for which the interepidemic interval has been long (previous epidemics in 1981 and 1988). Search-frequency-based surveillance can never achieve the specificity of laboratory reporting. However, it can provide an effective early warning for infectious diseases in which the clinical syndromes are specific enough to prompt the clinician to search specific guidelines.

For Lyme disease, the increase and peak distribution of the database searches substantially preceded the increase and peak in the laboratory reports of *B. burgdorferi* to the NIDR. The diagnosis of early Lyme disease, with its characteristic skin rash (6), is entirely clinical; serologic diagnosis can be made no earlier than 3 weeks after a tick bite. This delay could explain why the database searches peaked well before the NIDR reports. The earlier peak in database searches could also reflect a search for preventive measures during summer outdoor activities or for tick bite management before symptoms appear.

Dr. Jormanainen works as a consulting physician at the Health Care Division of the General Staff of Finnish Defense Forces, where his research interests include infectious disease epidemiology, surveillance, and public health issues.

### References

1. Hashimoto S, Murakami Y, Taniguchi K, Nagai M. Detection of epidemics in their early stage through infectious disease surveillance. Int J Epidemiol 2000;29:905-10.
2. Jousimaa J, Kunnamo I. PDRD-a computer-based primary care decision support system. Med Inform 1993;18:103-12.
3. Jousimaa J, Kunnamo I, Mäkelä M. Physicians' patterns of using a computerized collection of guidelines for primary health care. Int J Technol Assess Health Care 1998;14:484-93.
4. National Public Health Institute. Infectious diseases in Finland in 1995. Publications of the National Public Health Institute, KTL B8/1996. Helsinki: 1997.
5. Finnish Medical Society Duodecim. Available at: URL: *Http://www.ebm-guidelines.com*. [Note: The English version of the guidelines will be available for registered Internet users in 2001.]
6. Benenson AS, editor. Control of communicable diseases manual. 16th ed. Washington, DC: American Public Health Association; 1995.

## References

[PDF_00201] Hashimoto S., Murakami Y., Taniguchi K., Nagai M. (2000). Detection of epidemics in their early stage through infectious disease surveillance.. Int J Epidemiol.

[PDF_00206] Jousimaa J., Kunnamo I., Mäkelä M. (1998). Physicians' patterns of using a computerized collection of guidelines for primary care.. Int J Technol Assess Health Care.

[PDF_00204] Jousimaa J., Kunnamo I. (1993). PDRD--a computer-based primary care decision support system.. Med Inform (Lond).

